# On-Chip TaO*_x_*-Based Non-volatile Resistive Memory for *in vitro* Neurointerfaces

**DOI:** 10.3389/fnins.2020.00094

**Published:** 2020-02-26

**Authors:** Maksim Zhuk, Sergei Zarubin, Igor Karateev, Yury Matveyev, Evgeny Gornev, Gennady Krasnikov, Dmitiry Negrov, Andrei Zenkevich

**Affiliations:** ^1^Laboratory of Functional Materials and Devices for Nanoelectronics, Moscow Institute of Physics and Technology, Moscow, Russia; ^2^National Research Center, Kurchatov Institute, Moscow, Russia; ^3^Deutsches Elektronen-Synchrotron, Hamburg, Germany; ^4^Molecular Electronics Research Institute (MERI), Moscow, Russia; ^5^Laboratory of Neurocomputing Systems, Moscow Institute of Physics and Technology, Moscow, Russia

**Keywords:** neural tissue, *in vitro* neurointerfaces, high-density microelectrode arrays, non-volatile memory, resistive switching, tantalum oxide, 1T-1R device, back-end-of-line process

## Abstract

The development of highly integrated electrophysiological devices working in direct contact with living neuron tissue opens new exciting prospects in the fields of neurophysiology and medicine, but imposes tight requirements on the power dissipated by electronics. *On-chip* preprocessing of neuronal signals can substantially decrease the power dissipated by external data interfaces, and the addition of embedded non-volatile memory would significantly improve the performance of a co-processor in real-time processing of the incoming information stream from the neuron tissue. Here, we evaluate the parameters of TaO*_x_*-based resistive switching (RS) memory devices produced by magnetron sputtering technique and integrated with the 180-nm CMOS field-effect transistors as possible candidates for on-chip memory in the hybrid neurointerface under development. The electrical parameters of the optimized one-transistor–one-resistor (1T-1R) devices, such as the switching voltage (approx. ±1 V), uniformity of the *R*_off_/*R*_on_ ratio (∼10), read/write speed (<40 ns), and the number of the writing cycles (up to 10^10^), are satisfactory. The energy values for writing and reading out a bit ∼30 and ∼0.1 pJ, respectively, are also suitable for the desired *in vitro* neurointerfaces, but are still far too high once the prospective *in vivo* applications are considered. Challenges arising in the course of the prospective fabrication of the proposed TaO*_x_*-based RS devices in the back-end-of-line process are identified.

## Introduction

Modern electrophysiological techniques provide us with the versatile tools to study the inner workings of living neuronal circuits and open an opportunity to control them at the finest level. These methods, ranging from patch clamp to high-density microelectrode arrays, are of tremendous use in single-cell, neuronal culture, and brain studies (Bonifazi and Fromherz, 2002; Eversmann et al., 2011; Eickenscheidt et al., 2012; Szostak et al., 2017; Luan et al., 2018). Moreover, the advances in electrophysiology and neuroscience provide the possibility to implement novel medical devices, such as neuroprosthetics and brain–computer interfaces. In turn, the technological development of microelectronics and microfabrication have made it possible to implement tiny devices that can simultaneously receive the data from tens of thousands of channels (Frey et al., 2010; Massobrio et al., 2015). When combined with modern data processing techniques, such as spike sorting algorithms, these capabilities can be used to handle the data describing the real-time behavior of thousands of neurons in living tissue.

Meanwhile, advanced neuron stimulation techniques, such as optogenetics, have emerged (Goncalves et al., 2017). Using such tools, one can create precise high-bandwidth bidirectional interfaces to neuronal tissue, which is of interest not only to fundamental neurophysiological studies but for pharmacology and medicine as well.

Direct contact with living tissue imposes tight requirements on the power dissipated by electronics. On the other hand, highly integrated electrophysiological devices provide tremendous amount of data, and in all cases, except for simple recording experiments, these data should be processed in real time. In this respect, off-loading raw data to remote processing equipment is not the best solution since, in high-density systems, the data acquisition rate can reach several gigabytes per second, and the data transfer circuitry will itself use a substantial amount of power. Moreover, this transfer and processing will introduce additional delays and, particularly in the case of medical applications, it is inconvenient to route such wide high-speed interfaces to the external processing devices. Today, it is possible to make processing devices with power requirements of less than 20 mW/GOPS (Reuther et al., 2019) and this paves the way to preprocess *on-chip* neuronal signals from several tens of thousands of channels. Considering a typical event rate of 1,000 events per second, observed by a probe of comparable area (Juavinett et al., 2019) and estimating the amount of computations required to classify a single event as 10^6^ operations, one can detect and classify neuronal spikes in less than a 100-mW power envelope, which is much less than it would be required to transmit the raw data out (around 300–400 mW) for serial link with the required bandwidth (Hsiao et al., 2006).

Nevertheless, even using on-chip processing of the raw data, it is still desirable to lower the power consumption of the data processing circuitry further down. One way to achieve this is to replace the static memories used by data processors with some kind of the emerging non-volatile memory. Since static memories are responsible for a substantial fraction of the dissipated power, this approach looks appealing.

**FIGURE 1:**
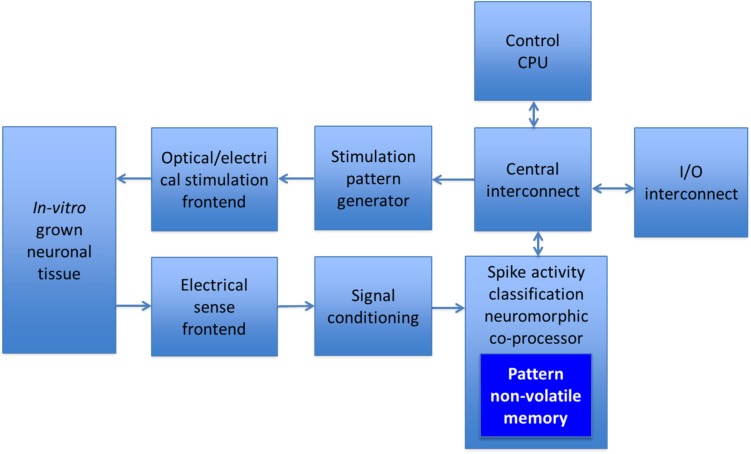
Schematic diagram of a high-density *in vitro* hybrid neurointerface for bidirectional communication with neuronal culture.

The above-mentioned approaches are currently being investigated, ultimately aiming at the development of a hybrid neurointerface for bidirectional communication with the living neuronal tissue in real time. The schematic diagram of such prospective neurointerface is shown in Figure 1. As pointed out above, the idea is to reduce the external input/output data rate and to enable online processing of neuronal activity. A substantial data rate reduction can be achieved by processing raw voltage waveforms to extract spiking activity from neurons in contact with the interface electrode array. Such procedure requires the application of data clustering algorithms, known as spike sorting, which work by matching raw data to pre-extracted encoded patterns and adapting to changes online. Although these patterns change slowly following the changes in the neuronal tissue as well as the electrical drift of electrodes, access to them is constantly required, and every extracted spike demands quite a large exchange with the memory. The detailed architecture of the neurointerface will be described elsewhere. The current work presumes that, potentially, the density of resistive random access memory (ReRAM) can be substantially higher than those of static RAM (SRAM). In addition, suggesting that the spiking activity of neurons occurs irregularly, the corresponding access to the memory is relatively rare, and since ReRAM in the retention mode does not require any (static) energy consumption, it would eventually be beneficial compared to SRAM. Therefore, here, we shall consider resistive switching (RS) non-volatile memory arrays, which can be integrated into the neurointerface chip. RS non-volatile memory has been previously used for processing-in-memory, particularly to simulate spiking networks (Pantazi et al., 2016; Wang et al., 2018), to accelerate vector–matrix multiplications (Prezioso et al., 2015) or to discriminate the recorded neuronal spiking events from the background activity and perform data compression of signals recorded by a multi-electrode array (Gupta et al., 2016).

Among the different non-volatile memory concepts, the one exploiting the reversible RS effect in thin films of transition metal oxides is a viable candidate (Rohde et al., 2005; Yang et al., 2010; Wong et al., 2012). The advantages of this kind of RS devices, once they are carefully optimized in terms of combination of materials, thickness of the functional layer, and switching pulses parameters, are as follows: good scalability (Govoreanu et al., 2011; Park et al., 2012), low power consumption (Goncalves et al., 2017), relatively high read/write speed (Lee et al., 2010, 2011), large number of writing cycles (Rohde et al., 2005; Kim et al., 2011), and, most importantly for the emerging neurointerfacing applications, the possibility to fabricate memory devices in the back-end-of-line (BEOL) process (Kim et al., 2012; Park et al., 2013; Goux et al., 2014; Li et al., 2018) of modern high-density mixed-signal fabrication flow. Dozens of papers have been published previously, describing the ReRAM devices employing mainly TiO_2_ (Frey et al., 2010; Jeong et al., 2009), HfO_2_ (Yang et al., 2010; Goncalves et al., 2017), and Ta_2_O_5_ (Rohde et al., 2005; Wedig et al., 2015) as functional layers. Over the last decade, very promising parameters have been demonstrated in terms of memory window, uniformity, endurance, and retention in RS devices integrated with the CMOS process, and ReRAM has been eventually successfully commercialized (see e.g. Fujitsu ReRAM memory data sheet)^1^. However, the variability of the electrical parameters for different transition metal oxide-based RS devices on the chip and from one switching cycle to another is still an issue, which is attributed to the inherent stochastic nature of the switching process (Fantini et al., 2014; Kim et al., 2014b). Also, the fact that such memory devices have been implemented does not necessarily imply they can be easily integrated into hybrid neurointerfaces under development. In particular, high-density embedded memory arrays should have RS devices placed in the lowest possible metallization layers to increase the density and to lower routing congestion. Moreover, this approach decreases the parasitic capacitances of memory lines, thus lowering the overall energy consumption. Such placement requires the stability of the RS device parameters upon subsequent processing steps, which are performed at temperatures up to ∼400°C as part of the standard fabrication technology (Walczyk et al., 2011).

Tantalum oxide is a popular functional layer used to devise resistive memory devices, and indeed, there have been a large number of published papers describing the operation of RS devices employing TaO*_x_* and different electrodes, including both 1-bit (Wong et al., 2012) and multi-bit (“analog”) (Kim et al., 2014a) switching behavior. The functional properties of transition metal oxide-based RS devices integrated with CMOS transistors (so-called one transistor–one resistor, 1T-1R, memory device) are well documented either (Lee et al., 2010, 2011). Nevertheless, once the goal is to fabricate TaO*_x_*-based 1T-1R RS devices to be used as built-in memory arrays for neurointerface applications, careful optimization of their parameters is needed so that they could fit the requirements, such as uniform switching voltage (in the range ±1–2 V), low energy consumption (∼10/0.1 pJ per write/read operation), modest retention time (several days), and high endurance (>10^7^ writing cycles).

In this work, we describe the implementation of TaO*_x_*-based resistive switching devices and their integration with the matrices of 180-nm CMOS transistors, ultimately aiming at the development of on-chip non-volatile memory arrays. Such memory can be used for the temporary storage of the data from the co-processor integrated on the bidirectional neurointerface chip and processing the information from the neuronal tissue in real time.

## Materials and Methods

**FIGURE 2:**
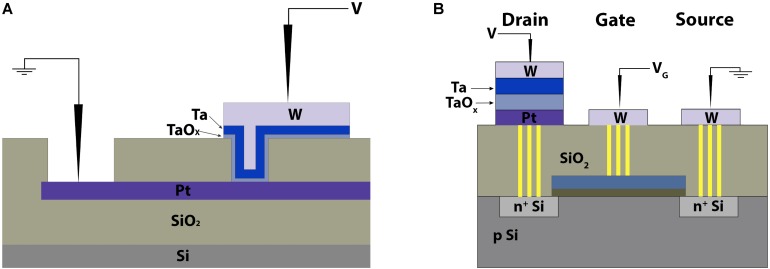
Schematic of panel (**A**) the Pt/TaO*_x_*/Ta 1R device topology with “cross-bar” geometry and (**B**) its integration with the field-effect transistor (FET).

Pt bottom electrode was deposited by magnetron sputtering. In order to form the metal–insulator–metal functional structure, windows ∼5 μm × 5 μm in size were first formed by dry plasma etching in a SiO_x_ layer grown plasma-enhanced chemical vapor deposition technique (the schematic is shown in Figure 2A). The TaO*_x_* layer, 5–20 nm in thickness, was deposited by direct current reactive magnetron sputtering of pure metal Ta target in pure O_2_. The top electrode (TE) Ta thin film with precise thickness in the nanometer range was further deposited in the same vacuum cycle from the same Ta target sputtered in an Ar atmosphere. To study the effect of the top electrode on the electrical properties of TaO*_x_*-based RS devices, alternative TEs, such as W, TiN, Ag, and Al, were also deposited (see Supplementary Table S1). The TE was capped with a thick W film in the same vacuum cycle to ensure the conductivity across the electrode area and protect the active Ta layer from oxidation.

Hard X-ray photoemission spectroscopy (HAXPES) analysis of as-grown TaO*_x_* films was performed at DESY synchrotron (endstation P22) at the excitation X-ray energy of *E* = 6 keV (an overall energy resolution of about 0.2 eV) with Specs 225-HV analyzer. The photoelectrons at such energies have the inelastic mean free path of ∼7 nm, thus increasing the probing depth up to ∼20–22 nm. Consequently, the relative contribution of the surface components is decreased, and true chemical composition across the bulk of the tantalum oxide layer can be revealed.

Sub-micron 1T-1R RS devices were fabricated by integrating the Pt/TaO*_x_*/Ta structures described above with 180-nm CMOS field-effect transistors in a 1,024 × 1,024 matrix (Figure 2B). Combining optical and e-beam lithography patterning, the RS devices were formed on top of remote W contacts to the drain of *n*-channel transistors (see Supplementary Figure S1a).

The electrical measurements were performed using a Keysight B1500A semiconductor device parameter analyzer in combination with a Cascade Microtech Summit 11000M probe station. The polarity of the voltage corresponds to the value on the top electrode. The forming voltage was derived from the first *I*–*V* curve. All endurance tests were performed by switching with square waveform voltage pulses in a vast time width range (40 ns–1 μs). For 1T-1R device characterization, an additional channel was used to control the gate voltage.

Transmission electron microscopy (TEM) study was performed with the S/TEM Titan 80–300 (Thermo Fisher Scientific) microscope equipped with a spherical aberration probe corrector, an energy-dispersive X-ray spectrometer (EDAX), and a high-angle annular dark-field detector (Fischione). The microscope was operated at 300 kV.

## Results and Discussion

**FIGURE 3:**
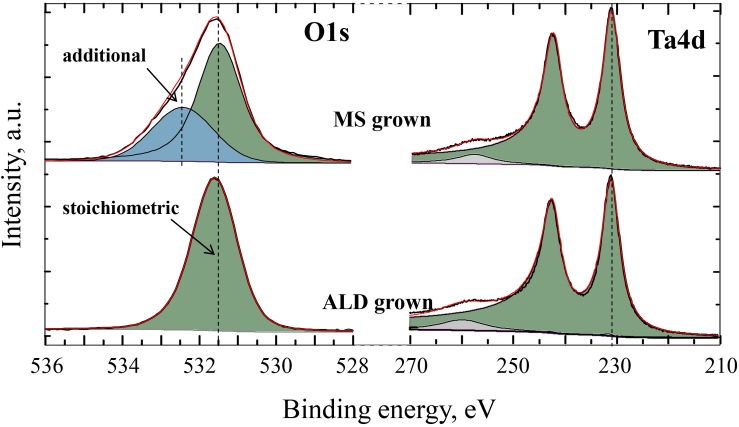
Core-level photoemission spectra of Ta 4d and O 1s lines of the 10-nm-thick TaO*_x_* layer as grown by magnetron sputtering obtained by the HAXPES technique.

The actual elemental composition of a few-nanometer-thick functional TaO*_x_* layer (capped with Al) is O:Ta ∼3.1, as revealed by Rutherford backscattering spectrometry (RBS) analysis (see Supplementary Figure S2). HAXPES analysis was used to confirm the overall super-stoichiometric elemental composition of the TaO*_x_* layer up to O:Ta ∼3.9, as compared to the stoichiometric Ta_2_O_5_ film grown by atomic layer deposition (spectra shown in Figure 3), implying a large excess of O atoms in the as-grown tantalum oxide layer. In addition, HAXPES data revealed two non-equivalent O states in the sputtered TaO*_x_* layer: the lines with BE = 532.3 eV and BE = 533.2 eV, which are attributed to the stoichiometric Ta_2_O_5_ (equivalent to that grown by ALD), and extra oxygen trapped during the sputtering process, respectively. By taking the relative Ta4d and O1s peak areas and using the corresponding photoeffect cross-sections, we calculated the overall composition to be Ta_1_O_3__.9_, in reasonable agreement with the RBS results.

**FIGURE 4:**
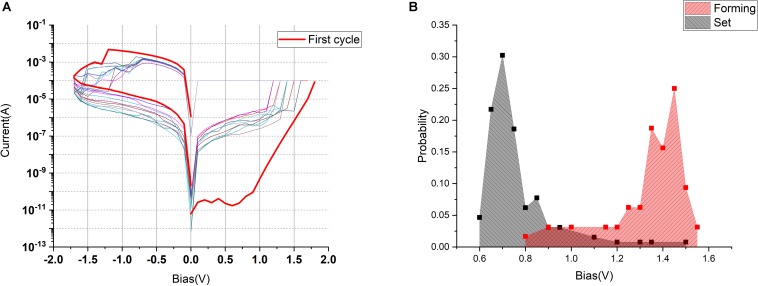
**(A)** Bipolar RS characteristics of the single Ta/TaO*_x_*/Pt device. **(B)** Normalized distribution of the forming (*red*) and SET (*black*) voltages for the Pt/TaO*_x_*/Ta 1T1R devices.

The fabricated Ta/TaO*_x_*/Pt RS cells were characterized in quasi-direct current (DC) mode by recording the *I*–*V* sweeps. Using the compliance current set at *I*_c_ = 10^–4^ A, the first switching cycle (called “electroforming”) was similar, within 0.5 V, to the subsequent ones, indicating the “forming-free” operation (Figures 4A,B). However, the variability of the switching parameters from cycle to cycle evident from the presented *I*–*V* curves is quite significant.

**FIGURE 5:**
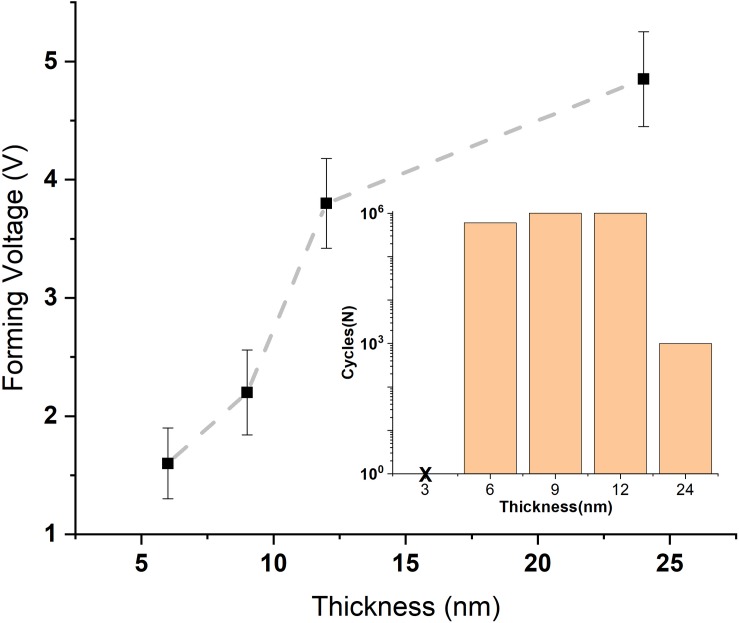
Plot of the electroforming voltage vs. the thickness of the functional TaO*_x_* layer in the Ta/TaO*_x_*/Pt RS 1R devices (*inset*: number of switching cycles for different TaO*_x_* layer thicknesses).

The thickness of the functional layer was further varied in the range of 3–24 nm in order to minimize the forming voltage while maintaining the maximal number of switching cycles. The data for *U*_form._ vs. the thickness of the TaO*_x_* layer are given in Figure 5. The number of the switching cycles for the devices with different thicknesses is given in the inset.

**FIGURE 6:**
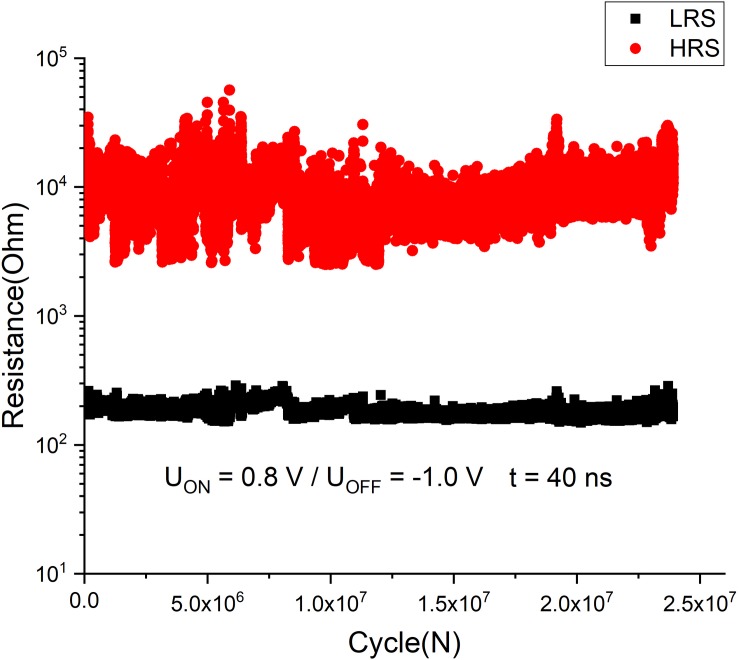
Endurance test of the “cross-bar” Ta/TaO*_x_*/Pt devices, switched by *U*_switch_. ∼ ± 1 V and *t* = 40 ns pulses.

Thus, an optimized functional structure was further used to fabricate devices in “cross-bar” geometry (Supplementary Figure S1b) for endurance tests with short (<50 ns) pulses. The results of such tests using *U*_on_ = 0.8 V/*U*_off_ = −1.0 V and *t* = 40 ns are presented in Figure 6.

**FIGURE 7:**
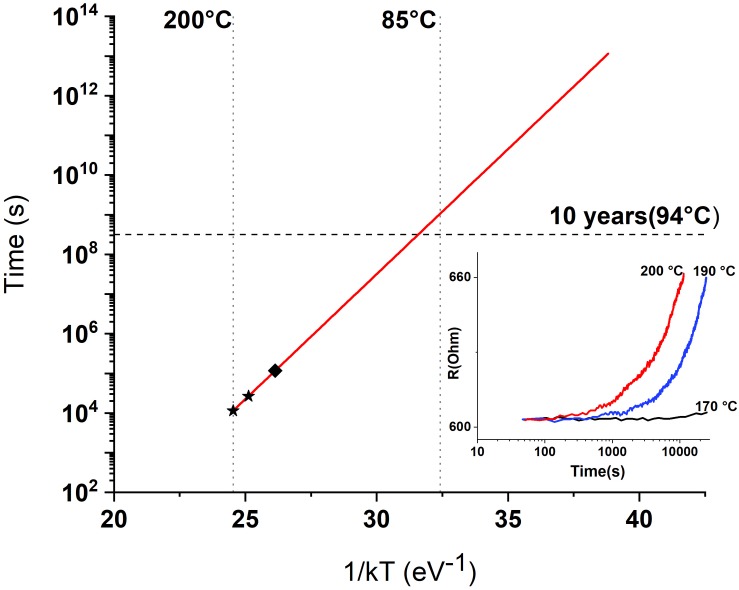
Arrhenius plot of the data retention properties for TaO*_x_*-based RS devices [*inset*: raw data on *R*_ON_(*t*) for different temperatures].

The retention test was further conducted for the same RS devices to examine their long-term memory functionality. After the SET process at room temperature, when all devices are in the low-resistance state (LRS), the chip is subjected to heating up to *T* = 200°C. The change of LRS *R*_on_ value by 10% was chosen as a criterion for the device unacceptable degradation. According to the Arrhenius plot of the measured data (Figure 7), the devices successfully pass the retention time of 10 years at *T* = 85°C. However, this temperature obviously cannot be reached in our application since on-chip memory matrix basically contacts the living neuron cells. Therefore, the operating temperature should be less than 40°C, which will be ensured by the heat removal in the current version of the *in vitro* chip. Direct simulation of the heating balance in the entire system with realistic contributions has not been carried out so far.

**FIGURE 8:**
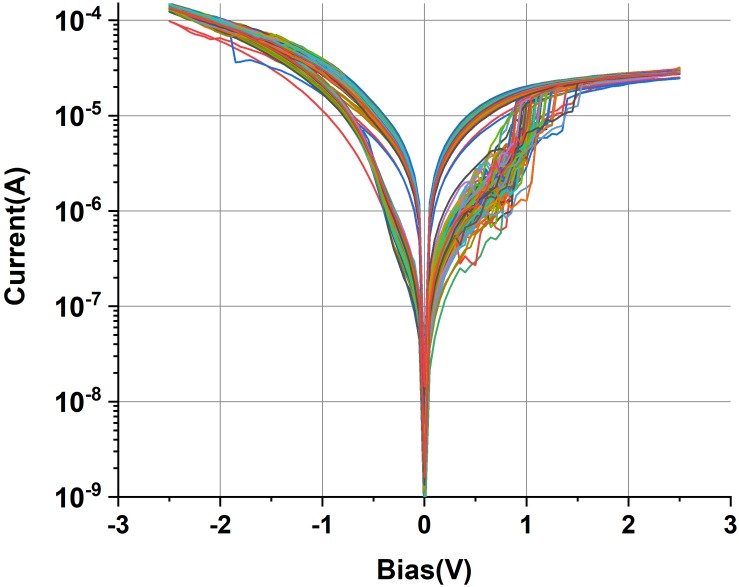
Sequential DC *I*–*V* curves of 1T-1R devices based on the Ta/TaO*_x_*/Pt stack.

**FIGURE 9:**
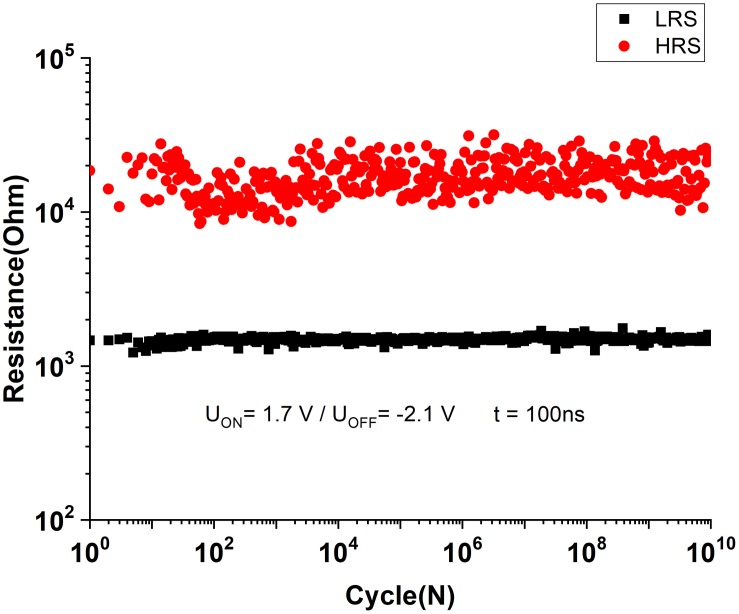
Number of writing cycles for the sub-micrometer Ta/TaO*_x_*/Pt crossbar RS devices.

Let us now describe the electrical properties of the 1T-1R RS devices employing the optimized 1R devices described above and the factory 180-nm CMOS transistors. While setting the compliance current by the gate voltage on the transistor at *I*_c_ = 3 × 10^–2^ mA, the average electroforming voltage of the 1T-1R devices in DC mode was less than 1.5 V, with stable *I*–*V* form during 100 DC cycles (Figure 8). The endurance test was performed by applying 100-ns-long switching pulses of selected memory cells. In order to maintain the best switching uniformity during the endurance test, the voltage pulse parameters were chosen to provide *R*_on_/*R*_off_ ∼10 and were set as *U* = + 1.7 V/−2.1 V, *t* = 100 ns. The fabricated sub-micrometer 1T-1R devices survive more than 10^10^ switching cycles without any signs of degradation (Figure 9).

**FIGURE 10:**
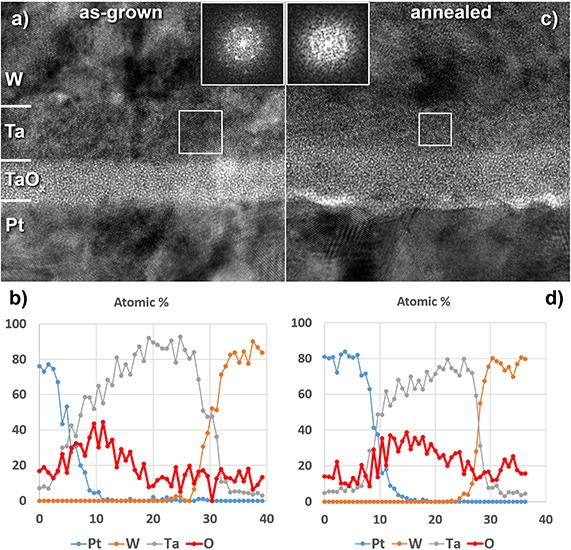
High-resolution TEM images of the Ta/TaO*_x_*/Pt device stack cross-section **(a)** and the same device following the annealing at *T* = 400°C, 30 min **(c)** (FFT of the Ta metallic layer is shown in the *inset*). **(b,d)** Elemental profiles across the stack as revealed by EDX analysis of the as-prepared and annealed stacks, respectively.

The use of 1T-1R built-in memory devices prepared in the BEOL process implies that they are fabricated early BEOL flow in a second–third metallization layer and should maintain their characteristics following the subsequent annealing at *T* = 400°C, which is part of the upper Al metallization layers technology. In order to investigate the compatibility of the fabricated 1T-1R memory devices with the 180-nm CMOS technology, they were post-annealed in vacuum (10^–6^ Torr) at *T* = 400°C for ∼30 min. It was found that, upon such annealing step, both pristine and electroformed devices have degraded, yielding very high leakage current and no resistive switching (Supplementary Figure S3). In order to investigate the degradation mechanism to possibly improve the thermal stability of the device parameters, we have used transmission electron microscopy analysis of the device stack cross-section before and after annealing. The images shown in Figure 10 indicate that the crystalline structure of the Ta layer has vanished upon annealing, while the relative thickness of the Ta vs. TaO*_x_* layers has changed. This suggestion is confirmed by comparing fast Fourier transform (FFT) pictures of the Ta layer before and after annealing, shown in the insets in Figures 10A,C. The oxygen concentration profile across the stack obtained using energy-dispersive X-ray (EDX) analysis with a sub-nanometer exciting electron beam reveals the redistribution of oxygen atoms in the stack (Figures 10B,D), implying the redox reaction at the Ta/TaO*_x_* interface. The reduction of tantalum oxide may eventually result in the dramatic decrease of its resistivity, which is the cause of the degradation.

In order to overcome the latter problem of degradation of the 1T-1R device properties during the BEOL process, magnetron sputtering of the tantalum oxide layer was performed at *T* = 400°C (prior to Ta layer deposition at room temperature). Such step results in the “normalizing” of the stoichiometry of the TaO*_x_* layer to O/Ta ≈ 2.5, as confirmed by RBS analysis (not shown), and excludes further oxidation of the Ta layer on top. Thus, the prepared Ta/TaO*_x_*/Pt-based 1T-1R devices successfully survive the annealing at *T* = 400°C for 30 min, to yield at least ∼10^7^ of the switching cycles (tests are still in progress) (Supplementary Figure S4). However, it comes at the price of increasing the electroforming voltage up to *U*_form_. = + 2.5 V (as compared to *U*_form_. = + 1.4 V for non-annealed devices).

In conclusion, on-chip non-volatile memory may significantly improve the performance of the co-processor to be used for real-time processing of the information stream received from the neuron tissue in neurointerfaces under development. Among several candidates, resistive memory (ReRAM) is a viable option. We have evaluated the use of Ta/TaO*_x_*/Pt-based resistive devices produced by magnetron sputtering and integrated with the 180-nm CMOS field-effect transistors as a possible candidate for on-chip memory. While the electrical parameters of the optimized 1T-1R devices, such as switching voltage (approx. ± 1 V), uniformity of the *R*_off_/*R*_on_ ratio (∼10), read and write speed (<40 ns), and the number of the writing cycles (∼10^10^), are encouraging, there are still challenges to overcome. In particular, the energy per write operation is ∼30 pJ, which is still much too high for use *in vivo* applications, where the power consumption and heat dissipation are critical constraints. Also, the perspective to fabricate memory on-chip in the BEOL process implies the metallization annealing steps (at *T* = 400°C), which affects the operation of the fabricated devices. Further work is necessary to optimize the device stack and fabrication technology to enable TaO*_x_*-based non-volatile memory matrices to be used in hybrid neurointerfaces under development.

## Data Availability Statement

All datasets generated for this study are included in the article/Supplementary Material.

## Author Contributions

MZ fabricated devices and performed electrical characterization and wrote the draft of the manuscript. SZ prepared samples and performed preliminary TEM analysis. IK analyzed with HR TEM/EDX the composition/structure of RS devices. YM analyzed with HAXPES the chemical composition of TaOx layers. EG and GK provided wafers with CMOS transistors pre-fabricated for further integraion of memory devices under investigation. DN motivated the work, analyzed the data and partially wrote the manuscript. AZ posed the problem, directed the experiments and contributed to the writing of the manuscript.

## Conflict of Interest

The authors declare that the research was conducted in the absence of any commercial or financial relationships that could be construed as a potential conflict of interest.

## Footnotes

1 https://www.fujitsu.com/global/documents/products/devices/semiconductor/memory/reram/MB85AS4MT-DS501-00045-1v0-E.pdf
