# Supplementary material for: On-Chip TaOx-Based Non-volatile Resistive Memory for in vitro Neurointerfaces
Source: Front Neurosci. 2020 Feb 26;14:94. doi: 10.3389/fnins.2020.00094 (PMC7055297; doi:10.3389/fnins.2020.00094)
Supplement: Supplementary file 1 [file Data_Sheet_1.pdf]

In order to choose the optimal TE in the functional structure of RS device, different materials have been studied. The cumulative results on the electrical properties of devices with fixed TaO_x layer thickness after averaging over multiple measurements are collected in Table S1. The combination Ta/TaO_x/Pt has been chosen as the most appropriate.

Table S1. Electrical properties of RS devices with different TE materials.

Sample	U_{form}, V	U_{ON}, V	U_{OFF}, V	Endurance
Ta/TaO_x (12 nm)/Pt	2.9±0.5	1.3 ± 0.3	-1.40 ± 0.25	10^5
W/TaO_x (12 nm)/Pt	> 5	-	-	-
TiN/TaO_x (12 nm)/Pt	1.3±0.2	0.8 ± 0.3	-1.3 ± 0.3	10^4
Ag/TaO_x (12 nm)/Pt	0.5±0.1	-	-	-
Al/ TaO_x (12 nm)/Pt	3.2±0.6	1.7 ± 0.2	-1.8 ± 0.2	$5 \cdot 10^3$

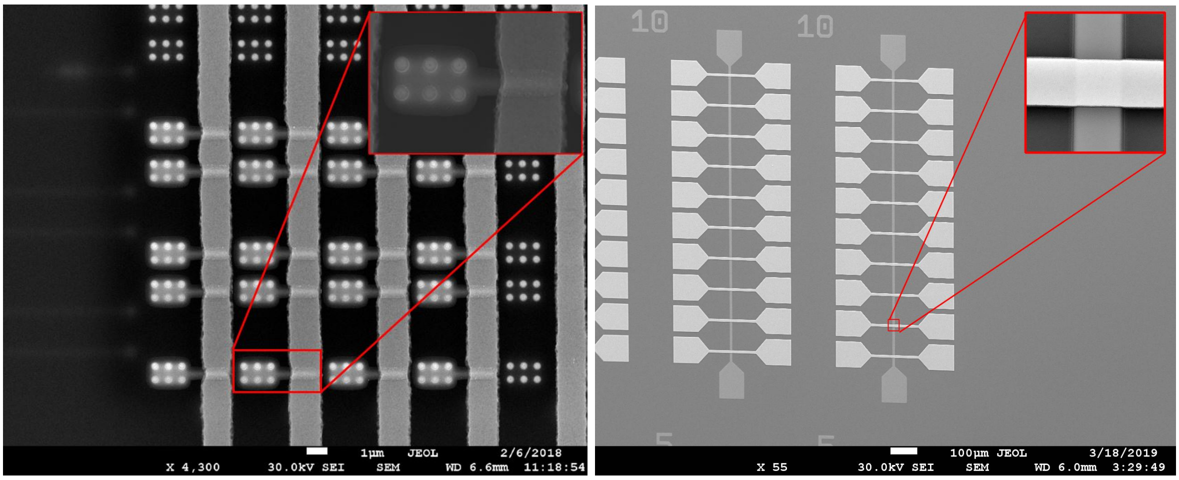

Figure S1. SEM image of: (a) 1024x1024 1T1R matrix “cross-bar” TaOx based RS devices integrated with 180 nm CMOS transistors; (b) single “cross-bar” 1R devices used for high-speed switching tests.

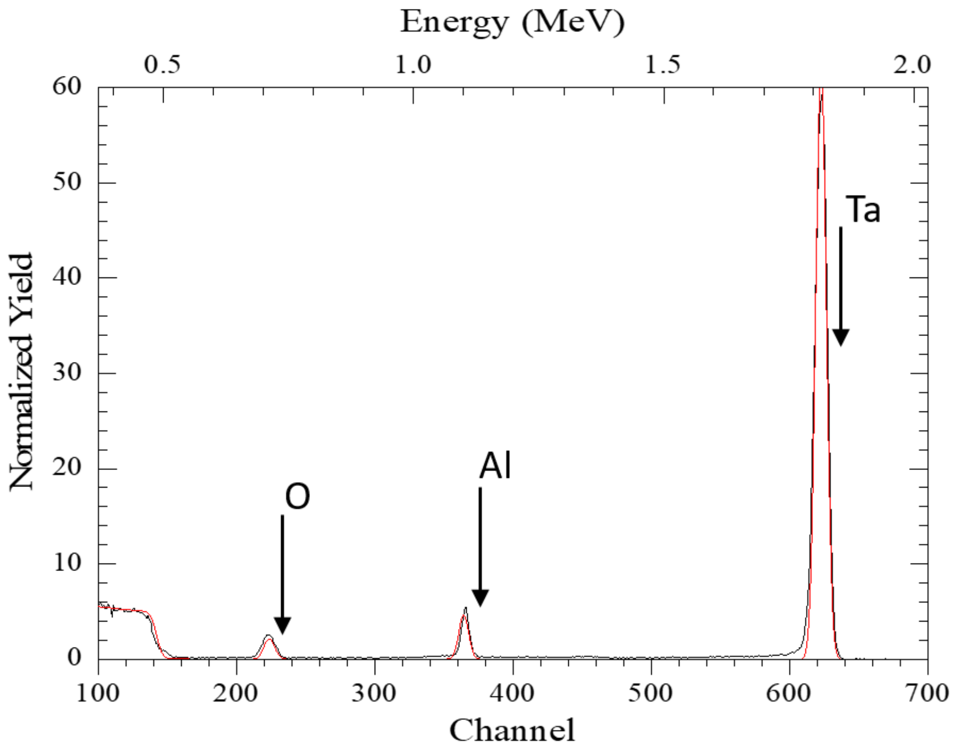

Figure S2. RBS spectrum of the magnetron sputtered TaOx functional layer used in the current work (black), modelled with the composition $Ta_1O_{3.1}$ (red). (The layer is deposited on the graphite substrate to increase the accuracy of O analysis, TaO_x was capped *in situ* with thin Al layer to exclude effects on the stoichiometry following the exposure to air).

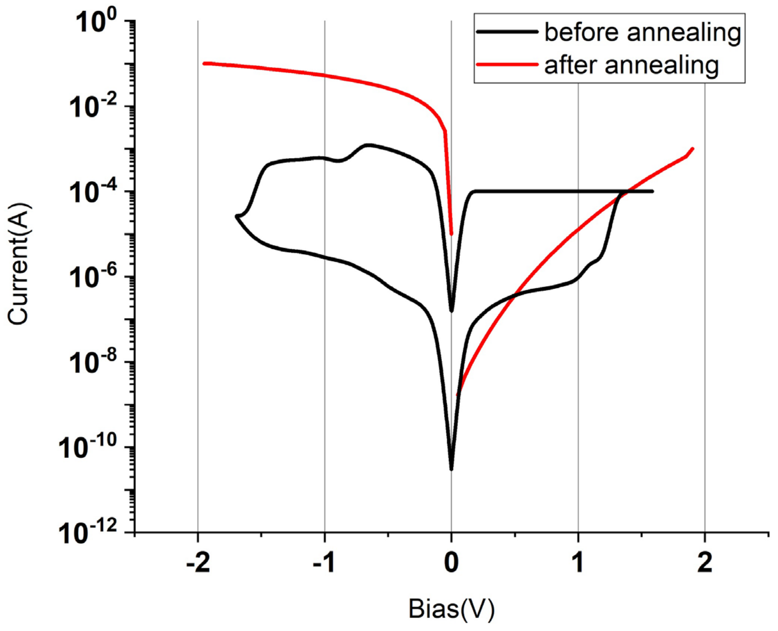

Figure S3. The comparison between DC I-V curves taken for 1R devices before and after annealing at T=400 C, 30 min.

In order to model the BEOL process following RS device fabrication, 1R devices were annealed in vacuum (10^{-6} Torr) at T=400°C for ~30 min. All memory cells have been found to degrade in terms of RS behavior, i.e. hard breakdown occurs while attempting to electroform them (note that the left branch of I-V curve has no “hysteresis effect”).

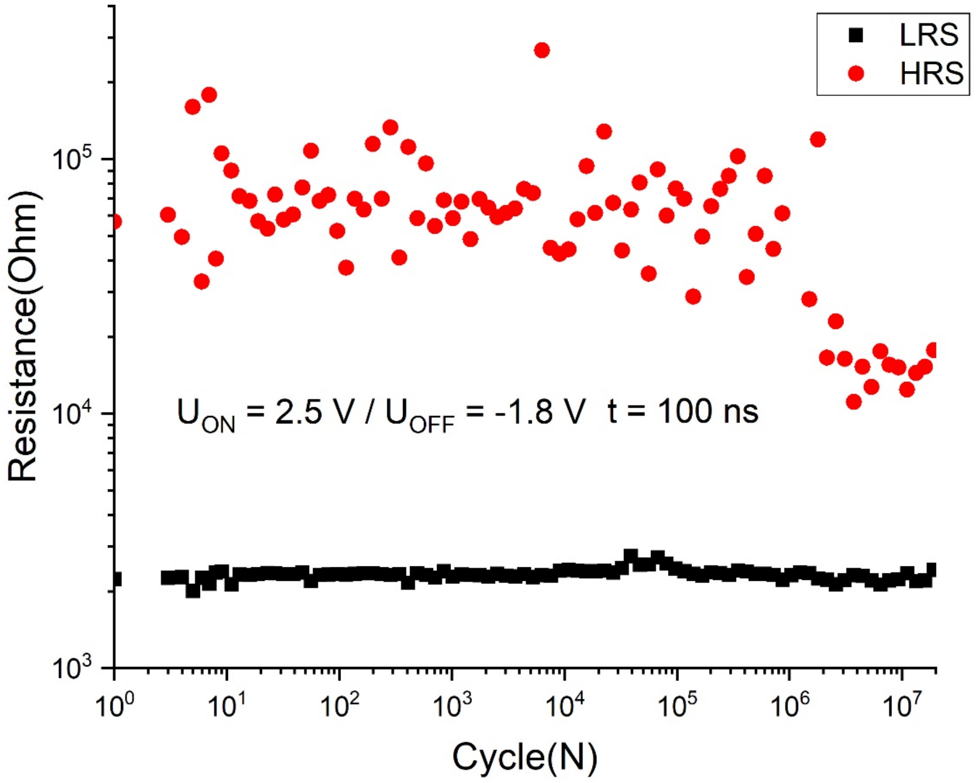

Figure S4. The endurance test for 1R Ta/TaO_x/Pt based devices with the modified parameters upon 400°C post-annealing in vacuum modelling BEOL process.
